# Automated Machine Learning for the Early Prediction of the Severity of Acute Pancreatitis in Hospitals

**DOI:** 10.3389/fcimb.2022.886935

**Published:** 2022-06-10

**Authors:** Minyue Yin, Rufa Zhang, Zhirun Zhou, Lu Liu, Jingwen Gao, Wei Xu, Chenyan Yu, Jiaxi Lin, Xiaolin Liu, Chunfang Xu, Jinzhou Zhu

**Affiliations:** ^1^ Department of Gastroenterology, The First Affiliated Hospital of Soochow University, Suzhou, China; ^2^ Department of Gastroenterology, The Changshu No. 1 Hospital of Soochow University, Suzhou, China; ^3^ Department of Obstetrics and Gynecology, The Second Affiliated Hospital of Soochow University, Suzhou, China

**Keywords:** automated machine learning, logistic regression analysis, severe acute pancreatitis, predictive models, artificial intelligence

## Abstract

**Background:**

Machine learning (ML) algorithms are widely applied in building models of medicine due to their powerful studying and generalizing ability. This study aims to explore different ML models for early identification of severe acute pancreatitis (SAP) among patients hospitalized for acute pancreatitis.

**Methods:**

This retrospective study enrolled patients with acute pancreatitis (AP) from multiple centers. Data from the First Affiliated Hospital and Changshu No. 1 Hospital of Soochow University were adopted for training and internal validation, and data from the Second Affiliated Hospital of Soochow University were adopted for external validation from January 2017 to December 2021. The diagnosis of AP and SAP was based on the 2012 revised Atlanta classification of acute pancreatitis. Models were built using traditional logistic regression (LR) and automated machine learning (AutoML) analysis with five types of algorithms. The performance of models was evaluated by the receiver operating characteristic (ROC) curve, the calibration curve, and the decision curve analysis (DCA) based on LR and feature importance, SHapley Additive exPlanation (SHAP) Plot, and Local Interpretable Model Agnostic Explanation (LIME) based on AutoML.

**Results:**

A total of 1,012 patients were included in this study to develop the AutoML models in the training/validation dataset. An independent dataset of 212 patients was used to test the models. The model developed by the gradient boost machine (GBM) outperformed other models with an area under the ROC curve (AUC) of 0.937 in the validation set and an AUC of 0.945 in the test set. Furthermore, the GBM model achieved the highest sensitivity value of 0.583 among these AutoML models. The model developed by eXtreme Gradient Boosting (XGBoost) achieved the highest specificity value of 0.980 and the highest accuracy of 0.958 in the test set.

**Conclusions:**

The AutoML model based on the GBM algorithm for early prediction of SAP showed evident clinical practicability.

## Introduction

Acute pancreatitis (AP) is a common cause of gastroenterology-related hospitalizations, with a morbidity rate of 34 per 100,000 individuals globally (Zhou et al., 2022). Although AP is inclined to be self-limiting, around 20% of patients will progress to severe AP (SAP), with persistent organ failure (POF) and poor prognosis (Wu et al., 2021). Therefore, early detection of SAP and early treatment such as fluid resuscitation are dispensable for reducing the morbidity and mortality of SAP.

Conventional scoring systems such as the RANSON score, bedside index of severity in acute pancreatitis (BISAP), modified computed tomography severity index (MCTSI), and Acute Physiology and Chronic Health Evaluation (APACHE) II have been generally applied to assess the severity of AP (Bollen et al., 2012; Mounzer et al., 2012). Some novel point systems, such as SABP (Hong et al., 2019), the pancreatic activity scoring system (PASS), and the Chinese simple scoring system (CSSS) (Wu et al., 2021), have been proposed in recent years. However, the traditional scores are relatively complicated for clinical use, and the novel scores are not generalized, whose ability to predict SAP varies and accuracy ranges from 0.70 to 0.95 (Hong et al., 2019; Paragomi et al., 2021; Wu et al., 2021).

Machine learning (ML) applied in medicine, both supervised and unsupervised, is becoming increasingly popular based on its efficient computing algorithms to learn from massive clinical data (Deo, 2015). Previous studies (Han et al., 2022; Liu et al., 2022; Xu et al., 2021; Yang et al., 2021; Zhang et al., 2021; Zhao et al., 2021; Zhou et al., 2021) have confirmed that ML has great potential in building models for disease diagnosis, prognosis prediction, survival analysis, etc. Traditional ML includes logistic regression (LR), support vector machine (SVM), random forest, etc. A novel ML called automated machine learning (AutoML) intelligently selects from various algorithms and hyperparameters to create models customized to target data. It takes less time to develop more accurate models using intelligent early stopping, cross validation, regularization, and hyperparameter optimization when compared to traditional ML.

Our study aims to train, validate, and test a series of ML models for early prediction of SAP within 72-h hospitalization using the H2O AutoML platform in multiple centers. Additionally, traditional logistic regression (LR) analysis is also developed, as well as four existing scoring systems.

## Methods

### Participants

A retrospective study was conducted in the three hospitals (the First Affiliated Hospital (First AFF), the Second Affiliated (Second AFF) Hospital, and Changshu No. 1 (Changshu) Hospital) of Soochow University from January 2017 to December 2021. Three hospitals are large-scale and fully equipped tertiary teaching hospitals in Suzhou, Jiangsu, China, There are 1,320 beds in Changshu Hospital, 2,050 beds in Second AFF, and more than 3,000 beds in First AFF. Changshu Hospital, as a county hospital, successfully established five major centers, including chest pain center, stroke center, atrial fibrillation center, etc. Second AFF is a tertiary level-A hospital integrating medicine, teaching, scientific research, prevention, and emergency care, with four research institutes and ten municipal key laboratories. First AFF, as one of the first grade-A hospitals under the Ministry of Health, ranked 32nd in the ranking of top hospitals in China in 2020. Data from two hospitals (First AFF and Changshu) were for development and internal validation, and data from another hospital (Second AFF) were for external testing.

**Figure 1:**
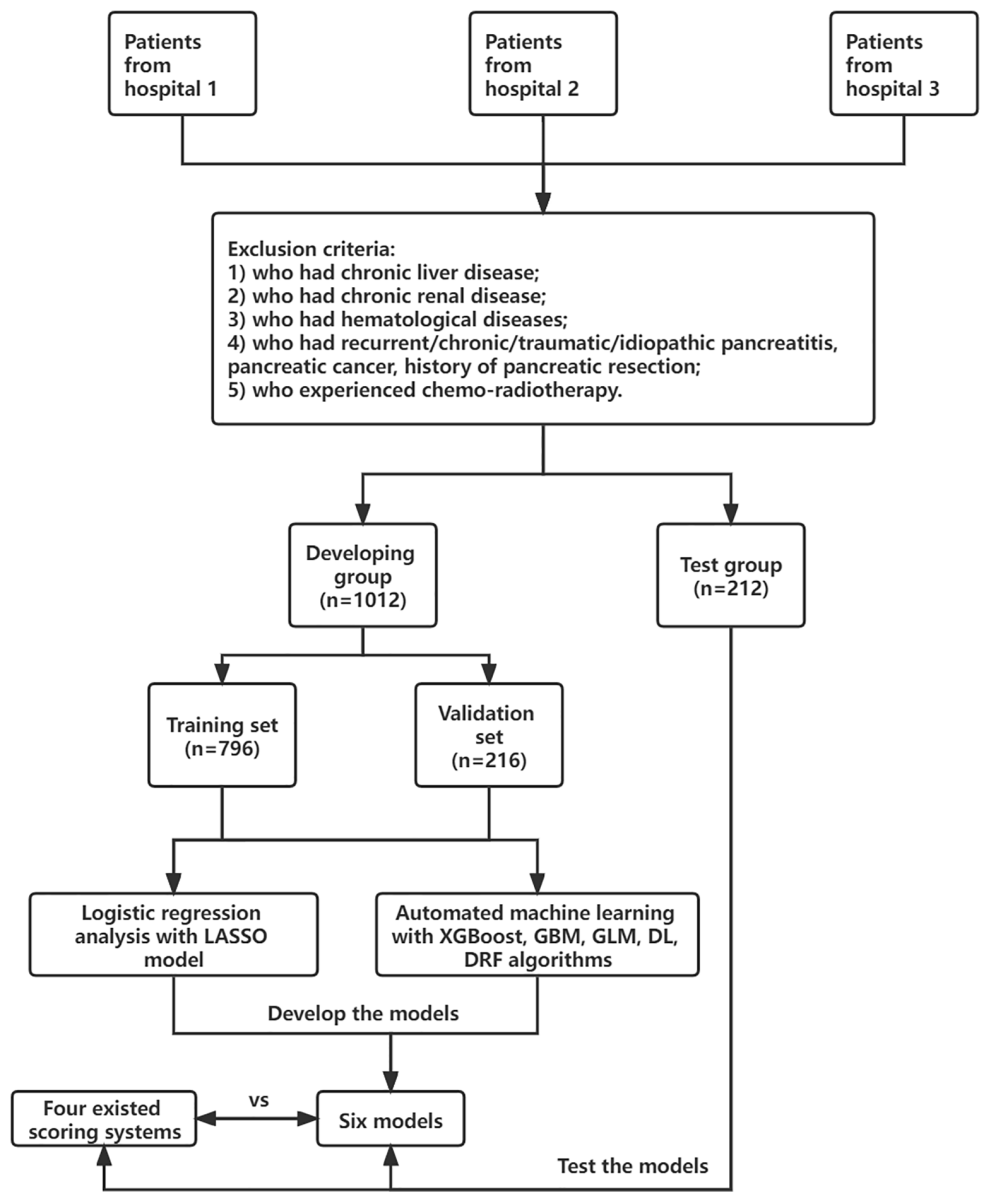
The flow chart of this study.

Adult patients (≥18 years old) who were diagnosed with AP based on the 2012 revised Atlanta classification of acute pancreatitis were enrolled. The diagnosis must meet at least two of the following criteria: (1) typical abdominal pain; (2) serum amylase beyond three times the upper limit of normal; and (3) images of characteristic findings of AP (Banks et al., 2013). Severe AP (SAP) was defined as AP with persistent organ failure (POF >48 h). Patients were divided into two groups: SAP and non-SAP. The exclusion criteria were patients who had chronic liver disease, chronic renal disease, hematological diseases, recurrent/chronic/traumatic/idiopathic pancreatitis, pancreatic cancer, and history of pancreatic resection; patients who experienced chemoradiotherapy; and patients who were pregnant. All patients were treated in accordance with the guidelines for the management of AP. This study was approved by the ethics committee of the First Affiliated Hospital of Soochow University (**Figure 1**).

### Data Collection

Demographic characteristics such as age, sex, smoke history, and clinical information such as etiology (biliary, hyperlipidemia, alcohol, and others) and concomitant diseases (hypertension and diabetes) were extracted from electronic medical records. Laboratory data within 24-h of admission were collected, including blood routine examination, coagulation tests, and serum biochemical tests. The presence of pleural effusion (PE) was recorded according to the computed tomography (CT) scan within 72-h of admission. Finally, a total of 41 variables were extracted for analysis. Details are listed in **Supplementary Table S2**. Missing variables, which were recognized as missing data at random, were multiple imputed using a random forest algorithm by the “mice” package of R software (Blazek et al., 2021). Other scoring systems such as systemic inflammatory response syndrome (SIRS), RANSON, MCTSI, BISAP, and SABP were calculated as described as Bollen et al. (2012), Hong et al. (2019) and Mounzer et al. (2012), if data were available. The flowchart of this study is shown in **Figure 1**.

### Logistic Regression

Univariate analysis was performed by the least absolute shrinkage and selection operator (LASSO) regression model with the “λ_1se” criterion in order to solve such multiple colinear relationships among the explanatory variables. A binary logistic backward stepwise regression analysis was used for model specification. The receiver operating characteristic (ROC) curve, the calibration curve, and the decision curve analysis (DCA) were applied to evaluate the predictive performance of our proposed model. A nomogram was constructed based on the independent risk factors identified in the multivariate analysis.

### Automated Machine Learning

The H2O package installed from the H2O.ai platform (www.h2o.ai) was applied to implement AutoML analysis, which automatically selects applicable algorithms and integrates them into multiple ensemble models. Algorithms include a default Random Forest (DRF), an Extremely Randomized Forest (XRF), a random grid of Gradient Boosting Machines (GBMs), a random grid of Deep Neural Nets (DLs), a fixed grid of Generalized Linear Models (GLMs), and a random grid of eXtreme Gradient Boosting (XGBoost). A 5-fold cross-validation grid search was performed on the training set for hyperparameter optimization, which was confirmed by evaluating AUCs for different combinations of hyperparameters included in the grid search. The confusion matrix, consisting of true positives (TP), true negatives (TF), false positives (FP), and false negatives (FN), was established to calculate sensitivity, specificity, positive predictive value (PPV), negative predictive value (NPV), positive likelihood ration (LR+), negative likelihood ration (LR−), accuracy (ACC), and areas under the ROC curve (AUCs) for evaluating discrimination performance of models. Formulas were as follows: ACC = (TP + TN)/(TP + FP + FN + TN); PPV = TP/(TP + NP); NPV = TN/(TN + FN); LR+ = sensitivity/(1-specificity); LR− = (1-sensitivity)/specificity. The visualization of AutoML was exhibited in the form of feature importance, SHapley Additive exPlanation (SHAP), and Local Interpretable Model Agnostic Explanation (LIME). SHAP analysis explained which features were most important for creating model predictions and how much they contributed to the overall model performance for a particular prediction (Bang et al., 2021). LIME analysis demonstrated how much each feature contributed to predicting the outcome by randomly giving examples from the test set.

### Statistical Analysis

Continuous variables were presented as mean ± standard deviation (SD) if fitting a normal distribution and as median (interquartile range) if not. Categorical variables were shown as frequencies. We compared the two groups by the Pearson Chi-square test or Fisher’s exact tests for categorical variables and the Student’s *t*-test or nonparametric Mann–Whitney *U* test for continuous variables. A two-sided *p* < 0.05 was considered statistically significant. The results were recorded as the odds ratio (OR) with corresponding 95% confidence intervals (CIs). R software (version 4.1.0) was used to implement all statistical analysis, including the H2O package (version 3.36.0.2), tableone package (version 0.12.0), tidyverse package (version 1.3.0), tidyquant package (version 1.0.2), and lime package (version 0.5.1).

## Results

### Demographic and Clinical Characteristics

A total of 1,224 patients were included in our study. SAP occurred in 136 cases (11.1%) in the whole cohort. Among all patients, 1,012 patients from two hospitals (First AFF and Changshu) were included in the developing dataset, and they were randomly split into the training and validation sets at a ratio of 8 to 2 (*n* = 796 in the training set and *n* = 216 in the validation set). In total, 212 patients from one hospital (Second AFF) were selected as a test dataset to evaluate model performance. In the developing dataset, 58.7% (594/1,012) were men and 41.3% (418/1012) were women. The median age was 52 years (IQR = 38–65 years) in the non-SAP group and 45.5 (IQR = 35–61.75 years) in the SAP group. In the test dataset, the onset of AP and SAP were also more commonly seen in male than in female patients and the median age ranged from 44 to 47 years. Consistent with what Xu et al. (2021) reported, biliary sludge or gallstones (39.49%) was the most frequent etiology of AP in our cohorts, followed by hypertriglyceridemia (17.87%). No statistical differences were observed in sex, age, smoke, history of hypertension, and diabetes in two groups of three datasets (*p* > 0.05). Details are listed in **Table 1**.

**Table 1:** Demographic and clinical characteristics of patients in training, validation and test groups.

Variables	The developing dataset (*n* = 1,012)				The test dataset (*n* = 212)		
	Group	Non-SAP (*n* = 888)	SAP (*n* = 124)	*p*-value	Non-SAP (*n* = 200)	SAP (*n* = 12)	*p*-value
Sex (%)	Male	518 (58.3)	76 (61.3)	0.597	133 (66.5)	9 (75.0)	0.770
	Female	370 (41.7)	48 (38.7)		67 (33.5)	3 (25.0)	
Age (year) (median [IQR])		52.00 [38.00, 65.00]	45.50 [35.00, 61.75]	0.141	47.00 [35.75, 65.25]	44.00 [33.75, 58.75]	0.810
Etiology (%)	Biliary	402 (45.3)	42 (33.9)	<0.001	88 (44.0)	5 (41.7)	0.461
	Hyperlipidemia	158 (17.8)	44 (35.5)		37 (18.5)	4 (33.3)	
	Alcoholic	48 (5.4)	5 (4.0)		21 (10.5)	0 (0.0)	
	Others	280 (31.5)	33 (26.6)		54 (27.0)	3 (25.0)	
Smoke (%)	No	767 (86.4)	108 (87.1)	0.936	161 (80.5)	9 (75.0)	0.927
	Yes	121 (13.6)	16 (12.9)		39 (19.5)	3 (25.0)	
Hypertension (%)	No	592 (66.7)	76 (61.3)	0.279	145 (72.5)	9 (75.0)	1.000
	Yes	296 (33.3)	48 (38.7)		55 (27.5)	3 (25.0)	
Diabetes (%)	No	773 (87.0)	102 (82.3)	0.187	170 (85.0)	8 (66.7)	0.202
	Yes	115 (13.0)	22 (17.7)		30 (15.0)	4 (33.3)	
MAP (mean (SD))		97.12 (11.95)	98.85 (15.57)	0.147	94.95 (12.59)	94.00 (14.20)	0.801
PLT (*10^9^/L) (mean (SD))		199.27 (66.43)	212.48 (79.94)	0.040	215.68 (66.31)	225.83 (84.44)	0.612
WBC (*10^9^/L) (median [IQR])		12.00 [9.16, 15.30]	16.07 [11.72, 20.64]	<0.001	11.90 [9.07, 14.83]	12.05 [10.50, 19.28]	0.216
*N* (*10^9^/L) (mean (SD))		10.35 (4.61)	14.45 (6.12)	<0.001	10.29 (4.68)	13.27 (6.26)	0.037
*L* (*10^9^/L) (median [IQR])		1.20 [0.80, 1.80]	0.91 [0.66, 1.50]	<0.001	1.30 [0.80, 1.80]	1.00 [0.75, 1.15]	0.174
NLR (median [IQR])		7.75 [4.32, 13.46]	13.71 [8.96, 23.31]	<0.001	6.87 [4.58, 13.60]	11.75 [7.25, 19.68]	0.060
HCT (L/L) (mean (SD))		0.47 (1.52)	0.83 (4.48)	0.097	0.86 (4.24)	0.46 (0.06)	0.741
RDW (%) (mean (SD))		13.00 (1.01)	13.27 (1.58)	0.009	12.92 (1.08)	12.76 (0.51)	0.602
Lr (%) (median [IQR])		10.85 [6.60, 17.30]	6.55 [3.98, 9.60]	<0.001	11.55 [6.47, 16.60]	7.85 [4.90, 11.43]	0.067
PCT (%) (mean (SD))		0.21 (0.11)	0.22 (0.08)	0.291	0.22 (0.06)	0.22 (0.07)	0.695
Cr (µmol/L) (median [IQR])		63.40 [53.90, 75.23]	61.50 [49.85, 85.85]	0.982	64.50 [54.00, 75.00]	66.50 [57.25, 106.75]	0.271
TB (µmol/L) (median [IQR])		21.00 [14.88, 32.23]	19.60 [12.83, 30.40]	0.165	17.15 [12.33, 26.15]	19.70 [14.20, 24.53]	0.666
DB (µmol/L) (median [IQR])		7.20 [4.50, 13.40]	7.35 [4.07, 12.95]	0.613	7.90 [5.47, 13.12]	10.20 [7.30, 16.95]	0.226
DTR (median [IQR])		0.36 [0.28, 0.48]	0.41 [0.30, 0.52]	0.042	0.47 [0.39, 0.60]	0.58 [0.48, 0.67]	0.058
Urea (mmol/L) (median [IQR])		4.90 [3.80, 6.20]	5.70 [4.27, 8.53]	<0.001	4.20 [3.38, 5.90]	6.35 [3.80, 13.40]	0.036
LDH (U/L) (median [IQR])		217.10 [178.00, 289.65]	341.00 [244.70, 498.88]	<0.001	199.00 [165.00, 250.25]	376.50 [211.75, 575.50]	0.001
Ca^2+^ (mmol/L) (mean (SD))		2.18 (0.19)	2.03 (0.28)	<0.001	2.10 (0.15)	1.73 (0.46)	<0.001
TG (mmol/L) (median [IQR])		1.42 [0.88, 3.30]	2.41 [1.27, 9.31]	<0.001	1.12 [0.66, 2.90]	3.42 [0.86, 9.17]	0.154
GLU (mmol/L) (median [IQR])		7.03 [5.84, 9.16]	8.18 [6.62, 11.79]	<0.001	6.98 [5.48, 9.27]	13.08 [9.50, 13.88]	0.002
TyG (median [IQR])		8.98 [8.41, 9.92]	9.79 [8.95, 11.11]	<0.001	8.79 [8.11, 9.75]	10.06 [9.05, 11.46]	0.031
ALT (U/L) (median [IQR])		39.10 [18.90, 141.00]	23.80 [14.20, 53.48]	<0.001	40.00 [17.75, 137.25]	24.00 [13.00, 55.75]	0.225
AST (U/L) (median [IQR])		30.00 [18.60, 86.25]	27.10 [18.95, 52.05]	0.129	28.00 [17.00, 68.75]	30.50 [20.00, 53.75]	0.919
GGT (U/L) (median [IQR])		86.40 [35.92, 267.00]	64.70 [27.75, 195.28]	0.09	102.50 [40.00, 266.00]	106.00 [61.75, 157.75]	0.959
ALP (U/L) (median [IQR])		90.00 [67.57, 131.85]	74.80 [55.80, 101.75]	<0.001	86.00 [67.00, 133.25]	75.50 [64.00, 84.50]	0.095
ALB (g/L) (mean (SD))		37.19 (4.99)	33.45 (6.47)	<0.001	38.17 (5.01)	33.46 (5.55)	0.002
K^+^ (mmol/L) (mean (SD))		4.01 (0.45)	3.99 (0.63)	0.256	4.12 (0.53)	4.32 (0.49)	0.205
AGR (median [IQR])		1.40 [1.20, 1.60]	1.20 [1.03, 1.40]	<0.001	1.50 [1.36, 1.69]	1.49 [1.20, 1.56]	0.266
PT (s) (mean (SD))		13.38 (2.19)	14.82 (3.05)	<0.001	13.95 (1.18)	14.80 (1.48)	0.018
INR (mean (SD))		1.10 (0.18)	1.22 (0.29)	<0.001	1.08 (0.11)	1.17 (0.12)	0.011
APTT (s) (mean (SD))		32.58 (6.82)	38.34 (18.43)	<0.001	37.74 (6.00)	40.48 (11.85)	0.154
CRP (median [IQR])		26.05 [3.49, 111.51]	149.02 [15.67, 265.12]	<0.001	76.95 [25.85, 143.78]	244.75 [103.62, 303.18]	0.003
CAR (median [IQR])		0.63 [0.09, 3.19]	4.09 [0.34, 8.99]	<0.001	2.04 [0.60, 4.06]	8.58 [3.07, 9.75]	0.001
RCR (median [IQR])		5.94 [5.53, 6.36]	6.57 [5.89, 7.24]	<0.001	6.06 [5.76, 6.54]	6.78 [6.01, 9.58]	0.019
SIRS (%)	No	640 (72.1)	30 (24.2)	<0.001	163 (81.5)	2 (16.7)	<0.001
	Yes	248 (27.9)	94 (75.8)		37 (18.5)	10 (83.3)	
PE (%)	No	609 (68.6)	15 (12.1)	<0.001	138 (69.0)	2 (16.7)	0.001
	Yes	279 (31.4)	109 (87.9)		62 (31.0)	10 (83.3)	
MCTSI (median [IQR])		2.00 [2.00, 4.00]	4.00 [4.00, 4.00]	<0.001	2.00 [2.00, 4.00]	5.00 [4.00, 6.00]	<0.001
RANSON (median [IQR])		1.00 [0.00, 2.00]	2.00 [1.00, 2.00]	<0.001	1.00 [0.00, 1.00]	2.00 [1.00, 2.25]	0.001
BISAP (median [IQR])		1.00 [0.00, 2.00]	2.00 [2.00, 3.00]	<0.001	1.00 [0.00, 1.00]	2.00 [1.25, 3.00]	0.002
SABP (median [IQR])		3.08 [-2.94, 10.83]	9.10 [1.43, 22.56]	<0.001	2.02 [-2.82, 7.33]	16.89 [-2.09, 28.61]	0.073

MAP, mean artery pressure; N, neutrophil; L, lymphocyte; NLR, neutrophil/lymphocyte; Lr, percentage of lymphocytes; Cr, creatinine; TB, total bilirubin; DB, direct bilirubin; DTR, direct bilirubin/total bilirubin; TG, total triglycerides; GLU, glucose; TyG, TG/GLU; AGR, albumin/globulin; CAR, CRP/albumin; RCR, RDW/Ca^2+^; ALB, albumin; SABP, acute biliary pancreatitis (0.55 + SIRS * 1.02 − 0.63 * ALB + 1.76 * BUN/0.356 + 1.66 * PE); PE, pleural effusion.

### Univariate and Multivariate Logistic Regression Analysis

**Figure 2:**
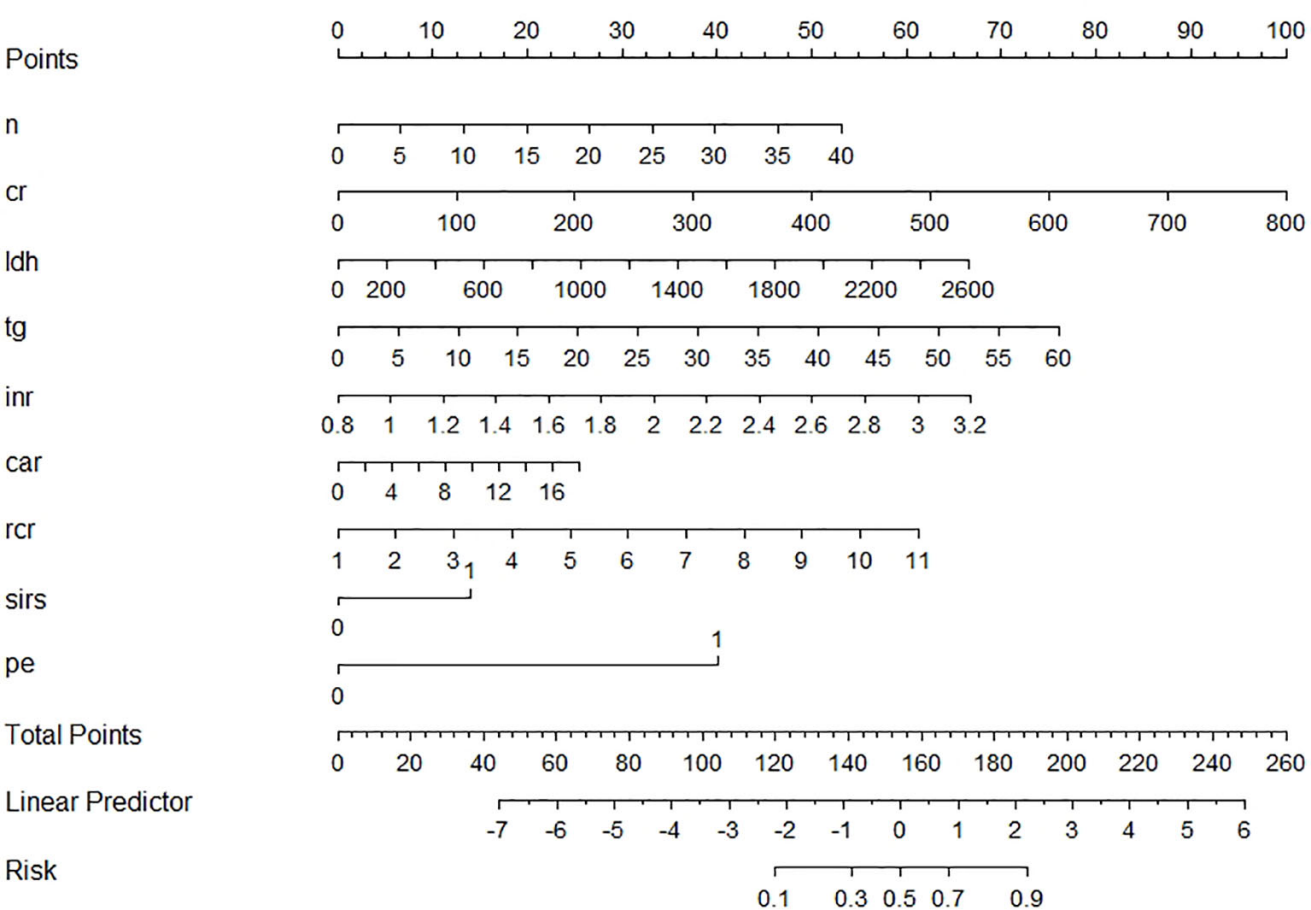
Nomogram of the LASSO model for the early prediction of severe acute pancreatitis.

Nine variables of the 41 variables were selected and later reserved as independent risk factors using the LASSO regression model with the “λ_1se (0.03)” criterion, which was achieved by 5-fold cross validation, to solve such multiple colinear relationships among the explanatory variables (**Supplementary Figure S1**). The final logistic model, including nine variables (neutrophil, creatinine, lactic dehydrogenase (LDH), total triglycerides (TGs), INR, ratio of red cell distribution width (RDW) to Ca^2+^ (RCR), ratio of CRP/albumin (CAR), SIRS, and PE), was developed as a nomogram and a score system for clinical use (**Figure 2**). The calibration curves of the training set, validation set, and test set are plotted in **Supplementary Figure S2**, and the mean absolute errors were 0.006, 0.033, and 0.036, respectively, demonstrating that the estimated risk using the LASSO model was close to the observed risk, indicating a high degree of reliability. The DCA plots of the training set, validation set, and test set are presented in **Supplementary Figure S3**, demonstrating that when the threshold probability of SAP predicted by the LASSO model was between 10% and 100%, an intervention might add more benefit (6%–10%). When a clinician considered the patient had a 10% chance of developing SAP, the patient might gain 4% of the benefit from an early intervention, according to the DCA of the test set, which is equivalent to detecting 4 SAP patients and suggesting zero unnecessary treatment per 100 patients. This is a direct comparison with treat none (the horizontal line in **Supplementary Figure S3**), which has zero true positives and zero false positives by default (Van Calster et al., 2018). The net benefit declares that the use of the LASSO model would improve patient outcome irrespective of patient or doctor preference. The ROC curve of the test set is presented in **Supplementary Figure S4**, and its AUC was 0.884 as shown in **Table 2**.

**Table 2:** Comparison of LR and AutoML models for early prediction of SAP in the test cohort.

	AUC	Sensitivity	Specificity	Accuracy	PPV	NPV	LR+	LR−
**AutoML**								
** GBM**	0.945	0.583	0.975	0.953	0.583	0.975	23.333	0.427
** XGBoost**	0.898	0.583	0.980	0.958	0.636	0.975	29.167	0.425
** DRF**	0.871	0.417	0.950	0.920	0.333	0.964	8.333	0.614
** GLM**	0.868	0.500	0.925	0.901	0.286	0.969	6.667	0.541
** DL**	0.860	0.500	0.920	0.896	0.273	0.968	6.250	0.543
**Logistic regression**								
** LASSO**	0.898	0.500	0.965	0.943	0.417	0.975	3.821	0.109
**Existed scoring systems**								
** RANSON**	0.764	0.667	0.800	0.896	0.188	0.954	3.335	0.416
** MCTSI**	0.869	1.000	0.588	0.611	0.128	1.000	2.427	0
** BISAP**	0.787	0.700	0.796	0.854	0.5	0.884	3.431	0.377
** SABP**	0.673	0.600	0.871	0.752	0.333	0.825	4.651	0.459

LR, logistic regression; AutoML, automated machine learning; SAP, severe acute pancreatitis; PPV, positive predictive value; NPV, negative predictive value; LR+, positive likelihood ration; LR−, negative likelihood ratio.

### Automated Machine Learning Analysis

**Figure 3:**
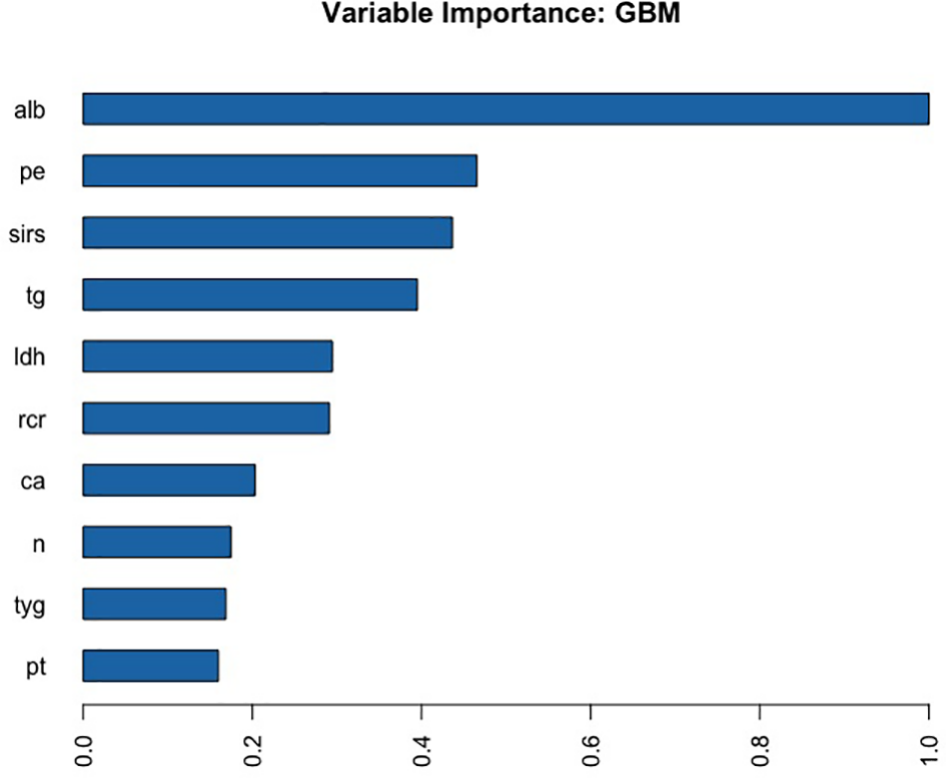
Variable importance of the GBM model in the training set, showing that albumin was the most important feature, followed by PE, SIRS, TGs, LDH, etc. ALB, albumin; PE, pleural effusion; SIRS, systemic inflammatory response syndrome; TG, triglyceride; LDH, lactic dehydrogenase; RCR, ratio of RDW to Ca^2+^; Ca, Ca^2+^; *n*, neutrophil count; TyG, ratio of triglyceride to glucose; PT, prothrombin time.

**Figure 4:**
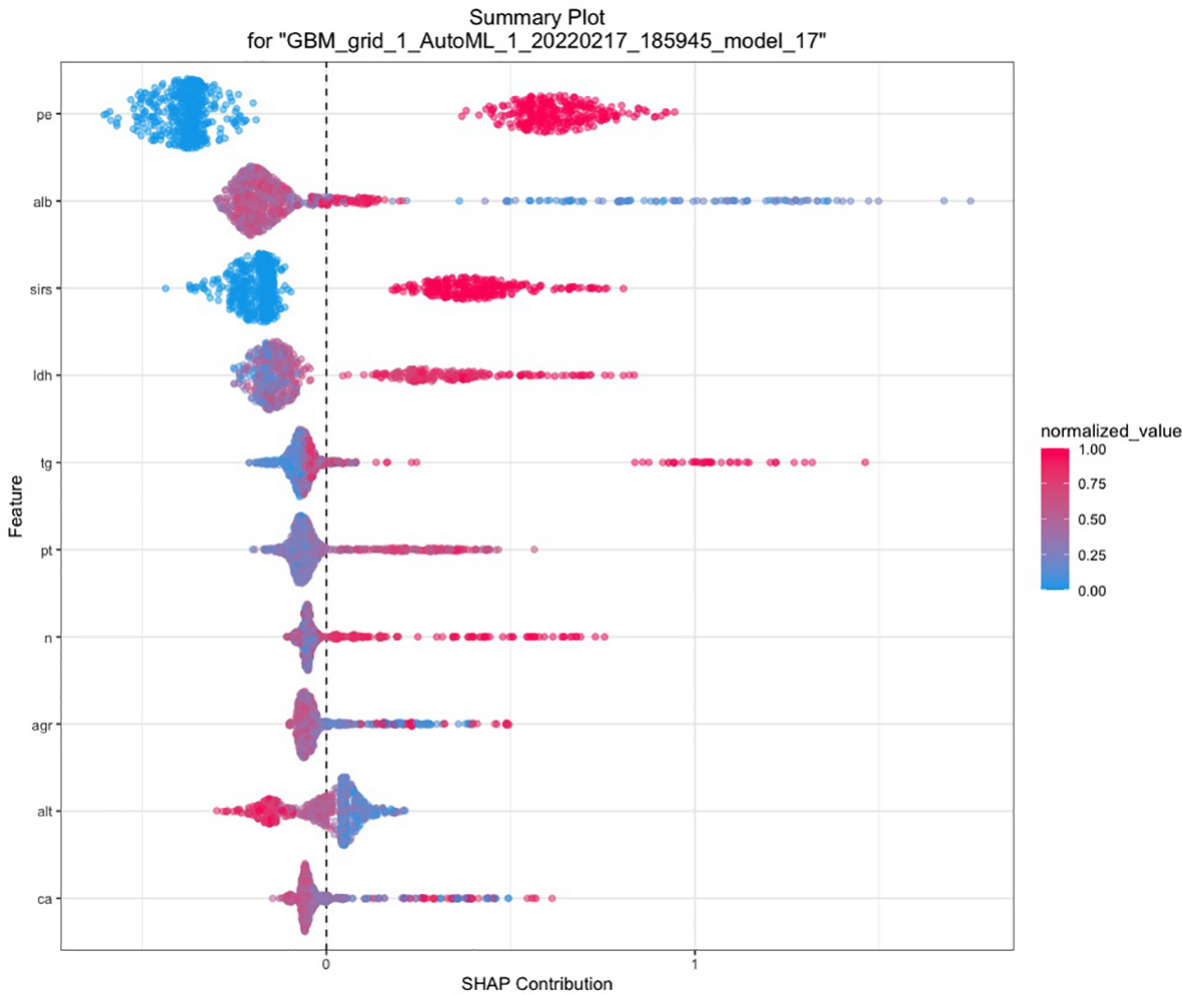
SHAP of the GBM model in the training set. The closer the values of the variables were to 1, the more likely patients were to progress to severity acute pancreatitis. SHAP, SHapley additive explanation; PE, pleural effusion; ALB, albumin; SIRS, systemic inflammatory response syndrome; LDH, lactic dehydrogenase; TG, triglyceride; PT, prothrombin time; n, neutrophil count; AGR, ratio of albumin to globulin; ALT, glutamic-pyruvic transaminase; Ca, Ca^2+^.

**Figure 5:**
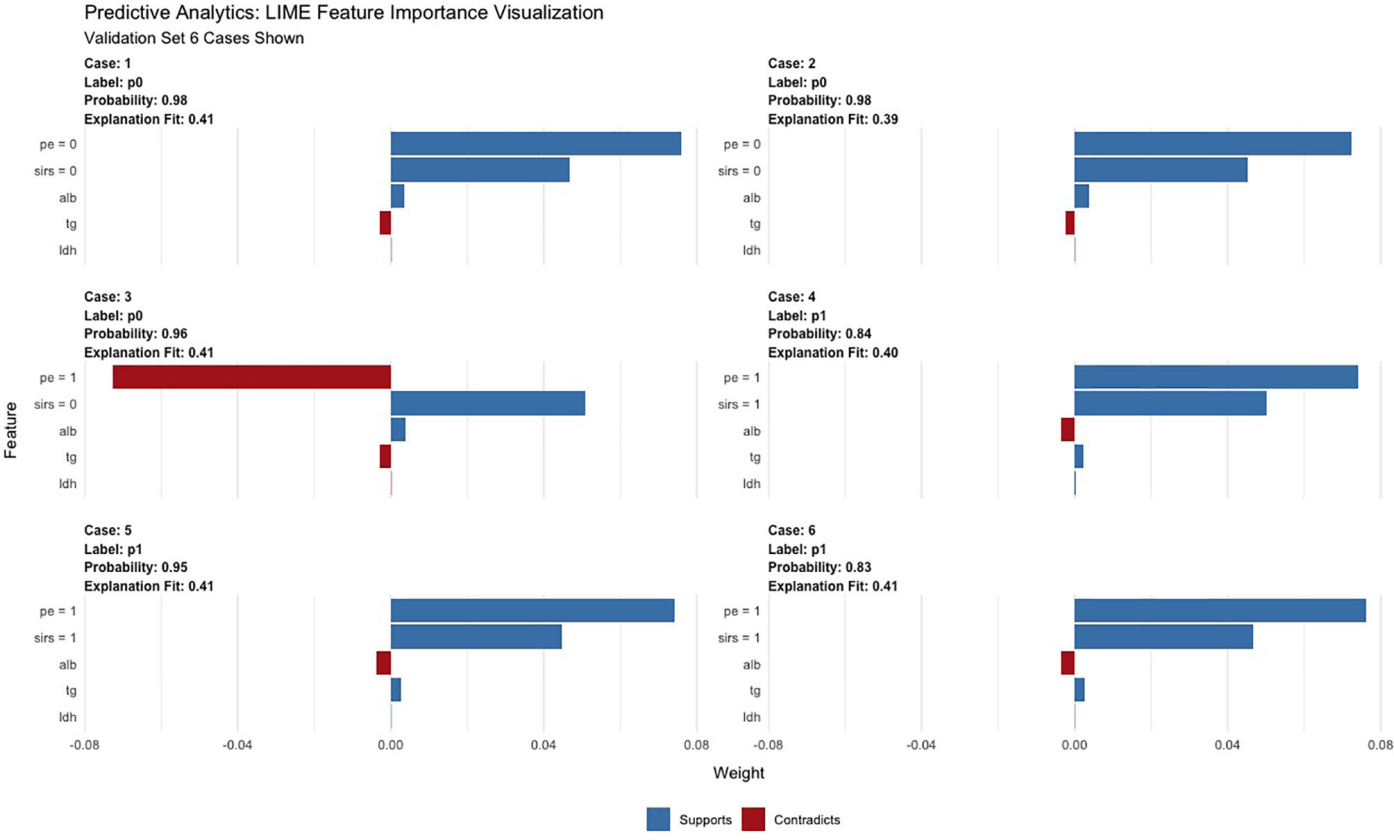
LIME of the GBM model in the test set. LIME, Local Interpretable Model Agnostic Explanation.

A total of 67 models were developed based on five machine learning algorithms (XGBoost, DL, GBM, GLM, and DRF), and stacked ensemble models were removed because of poor interpretability. The GBM model was the best among these models due to its highest value of AUC, which was a comprehensive evaluation for imbalanced samples. As shown in **Figure 3**, albumin was the most important feature, followed by PE, SIRS, TGs, LDH, RCR, Ca^2+^, neutrophil count, TyG, and prothrombin time (PT). Additionally, PE, neutrophil count, LDH, TGs, RCR, and SIRS were the important variables in common between the GBM model and the LASSO model. SHAP contribution plots based on GBM algorithms are presented in **Figure 4**, including ten important variables (PE, ALB, SIRS, LDH, TG, PT, neutrophil count, ratio of albumin to globulin (AGR), ALT, and Ca^2+^). The closer the values of the variables were to 1, the more likely patients were to progress to SAP. For example, the red part of PE which was concentrated on the right of axis = 0, revealed that the AP patient with PE would be more likely to develop SAP. **Table 2** demonstrates that GBM algorithm achieved the higher value of AUC than XGBoost, DRF, GLM, and DL algorithms (0.945, 0.898, 0.871, 0.868, and 0.860, respectively). The accuracy was 0.953, 0.958, 0.950, 0.925, and 0.920 according to the confusion matrix of GBM, XGBoost, DRF, GLM, and DL models on the test set. A LIME plot of the GBM model on the test set exhibited how several important variables contributed to the progress of SAP. As shown in **Figure 5**, for example, the case 4 had a high probability of 0.84 for progressing to SAP as predicted by the GBM model. PE was the most significant feature contributing to the prediction, followed by SIRS, TG, and LDH, while albumin had the opposite effect. The model can be deployed online, where clinicians can fill in the data in the table and then the predictive outcome will appear. Details can be seen on the official website (clouderizer.com).

### Comparisons Between Existing Scoring Systems and Models Developed by LR and AutoML

In general, the GBM and XGBoost models achieved the highest accuracy among these models, both beyond 0.950. The LASSO, DRF, and GLM models also obtained relatively high accuracy of 0.943, 0.920, and 0.901. The AUC values obtained by the ten models were 0.945 for GBM, 0.898 for XGBoost, 0.860 for DL, 0.868 for GLM, 0.871 for DRF, 0.898 for LR, 0.764 for RANSON, 0.787 for BISAP, 0.869 for MCTSI, and 0.673 for SABP. MCTSI achieved the highest sensitivity value of 1.000 and the lowest specificity of 0.588. XGBoost achieved the highest specificity value of 0.980 and the highest LR+ of 29.167.

## Discussion

In this study, we developed and tested several AutoML models to early identify who would progress to SAP. These models were all superior to existing scoring systems such as BISAP, RANSON, and MCTSI. Additionally, the GBM model obtained the highest value of AUC above 0.90, with specificity and accuracy all above 0.95.

Early prediction of SAP patients is essential for determining which patients require appropriate management such as intensive care, rapid fluid resuscitation, and early enteral nutrition (Gliem et al., 2021). Up to now, various scoring systems have been developed for early risk stratification of AP patients. Hong et al. (2019) built a prediction score called SABP, consisting of SIRS, albumin, blood urea nitrogen (BUN), and PE, which was trained and validated on 700 and 194 patients from two hospitals. The AUC of SABP on the external validation cohort in their study was superior to that in our study (0.873 vs. 0.673). This difference may be partly explained by the fact that BUN was not a routine examination within 24 h of admission in our hospital. Data deficiency may decrease the efficacy of the SABP score due to the reduction of sample size. Typical models such as RANSON, MCTSI, and BISAP in our study achieved inferior accuracy to the models we built. Besides the probability mentioned above, the other possible explanation may be that relevant indicators of those scores were categorical variables while were converted into continuous variables in our study, which may increase the probability of false-positive results due to the decrease in threshold value.

Compared with traditional univariate and sequent multivariate analyses, AutoML greatly improved work efficiency due to its less time consumption and higher accuracy. Additionally, ensemble models combined various machine-learning algorithms, utilizing multiclassifiers to predict the target outcome *via* taking a vote of individual predictions, which could enhance the overall performance (Goh et al., 2021). In this study, we selected four models built by five types of AutoML algorithms (GBM, XGBoost, DRF, GLM, and DL) for predicting the risk of SAP. All models, among which the GBM model ranking first in AUC and XGBoost and in accuracy on the test dataset, yielded satisfactory results. AUC gives a more feasible method to settle the problem of unbalanced data by putting the same weight on both classes in contrast to accuracy (Janitza et al., 2013). Additionally, since our aim is to early detect high-risk AP patients who would progress to SAP, sensitivity is a better choice, which is calculated as the ratio of subjects predicted positive with our proposed models to patients who are actually positive. Therefore, the GBM model was the best one in our study.

The SHAP analysis demonstrated that the occurrence of PE at admission was the most important feature for the GBM model. Yan et al. (2021) conducted research on pleural effusion volume (PEV) for predicting the severity of acute pancreatitis, with an AUC of 0.8158. Similarly, a study from Peng et al. in 2019 (Peng et al., 2020) also revealed that PEV holds a high accuracy (AUC = 0.839) for predicting the occurrence of SAP. In our study, PE was a common important feature selected not only by GBM but also by LASSO, BISAP, MCTSI, and SABP, indicating that PE and PEV were indeed reliable radiologic biomarkers in the prediction of SAP. Albumin has been proven as an independent risk factor for SAP according to previous studies (Hong et al., 2017; Hong et al., 2019; Wu et al., 2020; Xu et al., 2020). Hong et al. (2017) concluded that hypoalbuminemia within 24 h of hospital admission was greatly associated with increased probability of occurrence of POF and death in AP patients. A large-scale retrospective study analyzed the two open-access ICU databases to reveal the predictive significance of serum albumin in patients with AP (Xu et al., 2020). Chen and colleagues (Chen et al., 2021) carried out a subanalysis in hypertriglyceridemia pancreatitis populations for exploring the association between albumin and severity of AP. It was generally believed that elevated level of TG would drive the occurrence of SAP due to toxic effects on pancreatic acinar cell (Chen et al., 2021). The free fatty acids, hydrolyzed by pancreatic lipase from TGs, can bind to albumin in the serum and thus stimulate the inflammatory process. Therefore, Chen’s study effectively ruled out the confounding effect of TG and demonstrated that decrease of albumin was indeed an independent predictive factor. In our GBM model, albumin contributed the most to the predictive model, and TG ranked the fourth.

Zhang et al. (Peng et al., 2017) reported a significant correlation between the decrease of serum Ca^2+^ and the incidence of POF by triggering the SIRS process that recruits neutrophils and leads to further release of reactive oxygen species and organ damage. Another study from Gravito-Soares et al. (2018) proposed that RDW, a marker reflecting inflammation status, showed great predictive performance of AP severity with AUC of >0.810 and mortality with AUC of >0.842. Additionally, this study further suggested that RCR was an excellent predictor of AP severity with AUC value of 0.973. Han et al. (2021) also discovered a positive correlation between a high level of RCR and a poor prognosis for patients with AP. Consistent with the aforesaid studies, our study illustrated that Ca^2+^, RCR, neutrophil count, and SIRS were among the top 10 important variables in the GBM model.

A new scoring system for predicting organ failure in AP was proposed by Wu et al. (2020) in 2020, consisting of LDH, creatinine, albumin and Ca^2+^. LDH, also included in the typical RANSON score, is not commonly seen in recent proposed models. In 2008, Gurda-Duda et al. (2008) recommended LDH activity (within 12 h from disease onset) as a biomarker for early predicting prognosis of AP, with sensitivity of 63.6% and a specificity of 89.6%. It is well known that hypertriglyceridemia and hyperglycemia are correlated with severity of AP and organ failure (Gurda-Duda et al., 2008; Park et al., 2020; Chen et al., 2021). Gurda-Duda et al. (2008) suggested that blood glucose concentration (within 36 h from disease onset) could be a complementary measurement, with sensitivity of 72.7% and a specificity of 75.8%. Park et al. (2020) investigated the association between the TyG index (=ln [fasting TG (mg/dl) × fasting plasma glucose (mg/dl)]/2) and the severity of AP in 373 patients. The results showed that the TyG index not only accurately predicted SAP but also increased the predictive value of traditional models. The underlying mechanism might be explained by insulin resistance, which activated proinflammatory molecules accelerating the progression of SAP. PT, a parameter of coagulation state, was among the top 10 important features in our GBM model. However, a multivariate logistic regression analysis performed by Radenkovic et al. (2009) and three machine learning algorithms performed by Qiu et al. (2019) did not include these parameters into the final models.

Here, we built six predictive models using traditional logistic regression and AutoML, with high AUC of >0.860 and high accuracy of >0.896. Furthermore, it is more convenient and efficient to get the predictive probability for SAP using AutoML. Additionally, our study was a multicenter hospital-based research, which is a common way of efficiently evaluating a new technique and may provide a better foundation for the subsequent generalization of our models. However, there are some limitations in our study. Firstly, we divided AP patients into non-SAP and SAP instead of mild AP, moderate SAP, and SAP, which might decrease the sensitivity of our models. Secondly, our study is a retrospective study which might affect the performance of our models in a prospective clinical study. More prospective research needs to be conducted for external validation of our models. Thirdly, the online deployment website needs maintenance, and more data need to be inputted to improve the generalizability and performance of our models.

## Conclusion

We developed a series of effective models for early prediction of SAP based on AutoML platform, and these models outperformed the existing scoring systems, which might offer insights into AutoML applications in future medical studies. Additionally, the GBM model demonstrated practicable performance in early prediction better than LR and existing scoring systems.

## Data Availability Statement

The data presented in this study are available on request from the corresponding authors.

## Ethics Statement

This study was approved by the ethics committee of the first affiliated hospital of Soochow University. Written informed consent from the patients/ participants was not required to participate in this study in accordance with the national legislation and the institutional requirements.

## Author Contributions

MY and RZ contributed to data collection and writing. MY was responsible for statistical analysis. ZZ assisted in data collection and statistical analysis. LL, JG, and CY contributed to data cleaning and creating charts. JL and WX assisted in computer programming. XL and JZ contributed to revising this dissertation. JZ and CX managed this project and provided the funding. All authors listed have made a substantial, direct, and intellectual contribution to the work and approved it for publication.

## Funding

Science and Technology Plan of Suzhou City (SKY2021038) sponsored by CX. Youth Program of Suzhou Health Committee (KJXW2019001) sponsored by JZ.

## Conflict of Interest

The authors declare that the research was conducted in the absence of any commercial or financial relationships that could be construed as a potential conflict of interest.

The reviewer SL declared a shared parent affiliation with the author(s) MY, RZ, ZZ, LL, JG, WX, CY, JL, XLL, CX, JZ to the handling editor at the time of review.

## Publisher’s Note

All claims expressed in this article are solely those of the authors and do not necessarily represent those of their affiliated organizations, or those of the publisher, the editors and the reviewers. Any product that may be evaluated in this article, or claim that may be made by its manufacturer, is not guaranteed or endorsed by the publisher.

## Supplementary Material

Supplementary Table 1 Comparison of LR and AutoML models for early predicting SAP in the validation cohort. LR, logistic regression; AutoML, automated machine learning; SAP, severe acute pancreatitis.

Supplementary Figure 1 Penalty chart of predictive factors for severe acute pancreatitis based on LASSO regression analysis. Left: Regression coefficients. With the value of λ increasing, the absolute values of coefficients decrease. Right: Identification of the optimal λ value in the LASSO regression analysis was achieved by 5-fold cross-validation. (The left vertical line is drawn using the minimum criterion and the right vertical line is drawn using the 1_se criterion. In our study, LASSO regression model with ‘λ_1se’ criterion was used in the univariate analysis in order to solve such multiple co-linear relationships among the explanatory variables. LASSO, least absolute shrinkage and selection operator.

Supplementary Figure 2 Calibration curve of the LASSO model in the training, validation and test set, with the mean absolute errors being 0.006, 0.033 and 0.036, respectively. The calibration curves demonstrated that the estimated risk using LASSO model was close to the observed risk, indicating a high degree of reliability.

Supplementary Figure 3 Decision curve analysis of the LASSO model in the training, validation and test set. The DCA plots demonstrated that when the threshold probability of SAP predicted by the LASSO model was between 10% and 100%, an intervention might add more benefit (6-10%).

Supplementary Figure 4 ROC curves of all proposed models (GBM and LASSO models) and traditional scoring systems (BISAP, MCTSI, RANSON and SABP). GBM, Gradient Boost Machine; LASSO, the Least Absolute Shrinkage and Selection Operator; BISAP, bedside index of severity in acute pancreatitis; MCTSI, modified computed tomography severity index; RANSON, RANSON score; SABP[5].

## References

[B1] Bang C. S. Ahn J. Y. Kim J. H. Kim Y. I. Choi IJ and Shin W. G. (2021). Establishing Machine Learning Models to Predict Curative Resection in Early Gastric Cancer With Undifferentiated Histology: Development and Usability Study. J. Med. Internet Res. 23 (4), e25053. doi: 10.2196/25053 33856358 PMC8085749

[B2] Banks P. A. Bollen T. L. Dervenis C. Gooszen H. G. Johnson C. D. Sarr M. G.. (2013). Classification of Acute Pancreatitis–2012: Revision of the Atlanta Classification and Definitions by International Consensus. Gut 62 (1), 102–111. doi: 10.1136/gutjnl-2012-302779 23100216

[B3] Blazek K. van Zwieten A. Saglimbene V. Teixeira-Pinto A. (2021). A Practical Guide to Multiple Imputation of Missing Data in Nephrology. Kidney Int. 99 (1), 68–74. doi: 10.1016/j.kint.2020.07.035 32822702

[B4] Bollen T. L. Singh V. K. Maurer R. Repas K. van Es H. W. Banks PA and Mortele K. J. (2012). A Comparative Evaluation of Radiologic and Clinical Scoring Systems in the Early Prediction of Severity in Acute Pancreatitis. Am. J. Gastroenterol. 107 (4), 612–619. doi: 10.1038/ajg.2011.438 22186977

[B5] Chen L. Huang Y. Yu H. Pan K. Zhang Z. Man Y and Hu D. (2021). The Association of Parameters of Body Composition and Laboratory Markers With the Severity of Hypertriglyceridemia-Induced Pancreatitis. Lipids Health Dis. 20 (1), 9. doi: 10.1186/s12944-021-01443-7 33573658 PMC7879630

[B6] Deo R. C. (2015). Machine Learning in Medicine. Circulation 132 (20), 1920–1930. doi: 10.1161/CIRCULATIONAHA.115.001593 26572668 PMC5831252

[B7] Gliem N. Ammer-Herrmenau C. Ellenrieder V and Neesse A. (2021). Management of Severe Acute Pancreatitis: An Update. Digestion 102 (4), 503–507. doi: 10.1159/000506830 32422634 PMC8315686

[B8] Goh K. H. Wang L. Yeow A. Y. K. Poh H. Li K. Yeow JJL and Tan G. Y. H. (2021). Artificial Intelligence in Sepsis Early Prediction and Diagnosis Using Unstructured Data in Healthcare. Nat. Commun. 12 (1), 711. doi: 10.1038/s41467-021-20910-4 33514699 PMC7846756

[B9] Gravito-Soares M. Gravito-Soares E. Gomes D. Almeida N and Tome L. (2018). Red Cell Distribution Width and Red Cell Distribution Width to Total Serum Calcium Ratio as Major Predictors of Severity and Mortality in Acute Pancreatitis. BMC Gastroenterol. 18 (1), 108. doi: 10.1186/s12876-018-0834-7 29976140 PMC6034316

[B10] Gurda-Duda A. Kusnierz-Cabala B. Nowak W. Naskalski JW and Kulig J. (2008). Assessment of the Prognostic Value of Certain Acute-Phase Proteins and Procalcitonin in the Prognosis of Acute Pancreatitis. Pancreas 37 (4), 449–453. doi: 10.1097/MPA.0b013e3181706d67 18953261

[B11] Han T. Cheng T. Liao Y. He Y. Liu B. Lai Q.. (2022). Development and Validation of a Novel Prognostic Score Based on Thrombotic and Inflammatory Biomarkers for Predicting 28-Day Adverse Outcomes in Patients With Acute Pancreatitis. J. Inflammation Res. 15, 395–408. doi: 10.2147/jir.S344446 PMC8769056 35068938

[B12] Han T. Y. Cheng T. Liu B. F. He Y. R. Pan P. Yang W. T.. (2021). Evaluation of the Prognostic Value of Red Cell Distribution Width to Total Serum Calcium Ratio in Patients With Acute Pancreatitis. Gastroenterol. Res. Practice 2021, 6699421. doi: 10.1155/2021/6699421 PMC8331275 34354747

[B13] Hong W. Lillemoe K. D. Pan S. Zimmer V. Kontopantelis E. Stock S.. (2019). Development and Validation of a Risk Prediction Score for Severe Acute Pancreatitis. J. Transl. Med. 17 (1), 146. doi: 10.1186/s12967-019-1903-6 31068202 PMC6505180

[B14] Hong W. Lin S. Zippi M. Geng W. Stock S. Basharat Z.. (2017). Serum Albumin Is Independently Associated With Persistent Organ Failure in Acute Pancreatitis. Can. J. Gastroenterol. Hepatol. 2017, 5297143. doi: 10.1155/2017/5297143 29147647 PMC5632885

[B15] Janitza S. Strobl C. Boulesteix A. L. (2013). An AUC-Based Permutation Variable Importance Measure for Random Forests. BMC Bioinf. 14, 119. doi: 10.1186/1471-2105-14-119 PMC3626572 23560875

[B16] Liu J. Hu L. Zhou B. Wu C and Cheng Y. (2022). Development and Validation of a Novel Model Incorporating MRI-Based Radiomics Signature With Clinical Biomarkers for Distinguishing Pancreatic Carcinoma From Mass-Forming Chronic Pancreatitis. Trans. Oncol. 18, 101357. doi: 10.1016/j.tranon.2022.101357 PMC8818577 35114568

[B17] Mounzer R. Langmead C. J. Wu B. U. Evans A. C. Bishehsari F. Muddana V.. (2012). Comparison of Existing Clinical Scoring Systems to Predict Persistent Organ Failure in Patients With Acute Pancreatitis. Gastroenterology 142 (7), 1476–1482 quiz e1415–e1476. doi: 10.1053/j.gastro.2012.03.005 22425589

[B18] Paragomi P. Hinton A. Pothoulakis I. Talukdar R. Kochhar R. Goenka M. K.. (2022). The Modified Pancreatitis Activity Scoring System Shows Distinct Trajectories in Acute Pancreatitis: An International Study. Clin. Gastroenterol. Hepatol. 20 (6), 1334–1342.e4. doi: 10.1016/j.cgh.2021.09.014 34543736 PMC9060638

[B19] Park J. M. Shin S. P. Cho S. K. Lee J. H. Kim J. W. Kang C. D.. (2020). Triglyceride and Glucose (TyG) Index is an Effective Biomarker to Identify Severe Acute Pancreatitis. Pancreatology 20 (8), 1587–1591. doi: 10.1016/j.pan.2020.09.018 33008750

[B20] Peng T. Peng X. Huang M. Cui J. Zhang Y. Wu H and Wang C. (2017). Serum Calcium as an Indicator of Persistent Organ Failure in Acute Pancreatitis. Am. J. Emergency Med. 35 (7), 978–982. doi: 10.1016/j.ajem.2017.02.006 28291705

[B21] Peng R. Zhang L. Zhang Z. M. Wang Z. Q. Liu GY and Zhang X. M. (2020). Chest Computed Tomography Semi-Quantitative Pleural Effusion and Pulmonary Consolidation are Early Predictors of Acute Pancreatitis Severity. Quant. Imaging Med. Surg. 10 (2), 451–463. doi: 10.21037/qims.2019.12.14 32190570 PMC7063295

[B22] Qiu Q. Nian Y. J. Guo Y. Tang L. Lu N. Wen L. Z.. (2019). Development and Validation of Three Machine-Learning Models for Predicting Multiple Organ Failure in Moderately Severe and Severe Acute Pancreatitis. BMC Gastroenterol. 19 (1), 118. doi: 10.1186/s12876-019-1016-y 31272385 PMC6611034

[B23] Radenkovic D. Bajec D. Ivancevic N. Milic N. Bumbasirevic V. Jeremic V.. (2009). D-Dimer in Acute Pancreatitis: A New Approach for an Early Assessment of Organ Failure. Pancreas 38 (6), 655–660. doi: 10.1097/MPA.0b013e3181a66860 19436232

[B24] Van Calster B. Wynants L. Verbeek J. F. M. Verbakel J. Y. Christodoulou E. Vickers A. J.. (2018). Reporting and Interpreting Decision Curve Analysis: A Guide for Investigators. Eur. Urol. 74 (6), 796–804. doi: 10.1016/j.eururo.2018.08.038 30241973 PMC6261531

[B25] Wu H. Li J. Zhao J and Li S. (2020). A New Scoring System can be Applied to Predict the Organ Failure Related Events in Acute Pancreatitis Accurately and Rapidly. Pancreatology 20 (4), 622–628. doi: 10.1016/j.pan.2020.03.017 32273167

[B26] Wu Q. Wang J. Qin M. Yang H. Liang Z and Tang G. (2021). Accuracy of Conventional and Novel Scoring Systems in Predicting Severity and Outcomes of Acute Pancreatitis: A Retrospective Study. Lipids Health Dis. 20 (1), 41. doi: 10.1186/s12944-021-01470-4 33906658 PMC8080352

[B27] Xu X. Ai F. Huang M. (2020). Deceased Serum Bilirubin and Albumin Levels in the Assessment of Severity and Mortality in Patients With Acute Pancreatitis. Int. J. Med. Sci. 17 (17), 2685–2695. doi: 10.7150/ijms.49606 33162796 PMC7645339

[B28] Xu F. Chen X. Li C. Liu J. Qiu Q. He M.. (2021). Prediction of Multiple Organ Failure Complicated by Moderately Severe or Severe Acute Pancreatitis Based on Machine Learning: A Multicenter Cohort Study. Mediators Inflammation 2021, 5525118. doi: 10.1155/2021/5525118 PMC8112913 34054342

[B29] Yang D. J. Li M. Yue C. Hu WM and Lu H. M. (2021). Development and Validation of a Prediction Model for Deep Vein Thrombosis in Older non-Mild Acute Pancreatitis Patients. World J. Gastrointestinal Surg. 13 (10), 1258–1266. doi: 10.4240/wjgs.v13.i10.1258 PMC8554725 34754393

[B30] Yan G. Li H. Bhetuwal A. McClure M. A. Li Y. Yang G.. (2021). Pleural Effusion Volume in Patients With Acute Pancreatitis: A Retrospective Study From Three Acute Pancreatitis Centers. Ann. Med. 53 (1), 2003–2018. doi: 10.1080/07853890.2021.1998594 34727802 PMC8567956

[B31] Zhang D. Wang T. Dong X. Sun L. Wu Q. Liu J and Sun X. (2021). Systemic Immune-Inflammation Index for Predicting the Prognosis of Critically Ill Patients With Acute Pancreatitis. Int. J. Gen. Med. 14, 4491–4498. doi: 10.2147/ijgm.S314393 34413676 PMC8370754

[B32] Zhao B. Sun S. Wang Y. Zhu H. Ni T. Qi X.. (2021). Cardiac Indicator CK-MB Might be a Predictive Marker for Severity and Organ Failure Development of Acute Pancreatitis. Ann. Trans. Med. 9 (5), 368. doi: 10.21037/atm-20-3095 PMC8033390 33842589

[B33] Zhou Y. Ge Y. T. Shi X. L. Wu K. Y. Chen W. W. Ding Y. B.. (2022). Machine Learning Predictive Models for Acute Pancreatitis: A Systematic Review. Int. J. Med. Inform. 157, 104641. doi: 10.1016/j.ijmedinf.2021.104641 34785488

[B34] Zhou T. Xie C. L. Chen Y. Deng Y. Wu J. L. Liang R.. (2021). Magnetic Resonance Imaging-Based Radiomics Models to Predict Early Extrapancreatic Necrosis in Acute Pancreatitis. Pancreas 50 (10), 1368–1375. doi: 10.1097/mpa.0000000000001935 35041335

