# Supplementary material for: Automated Machine Learning for the Early Prediction of the Severity of Acute Pancreatitis in Hospitals
Source: Front Cell Infect Microbiol. 2022 Jun 10;12:886935. doi: 10.3389/fcimb.2022.886935 (PMC9226483; doi:10.3389/fcimb.2022.886935)
Supplement: Supplementary Table 1 — Comparison of LR and AutoML models for early predicting SAP in the validation cohort. LR, logistic regression; AutoML, automated machine learning; SAP, severe acute pancreatitis. [file DataSheet_1.docx]

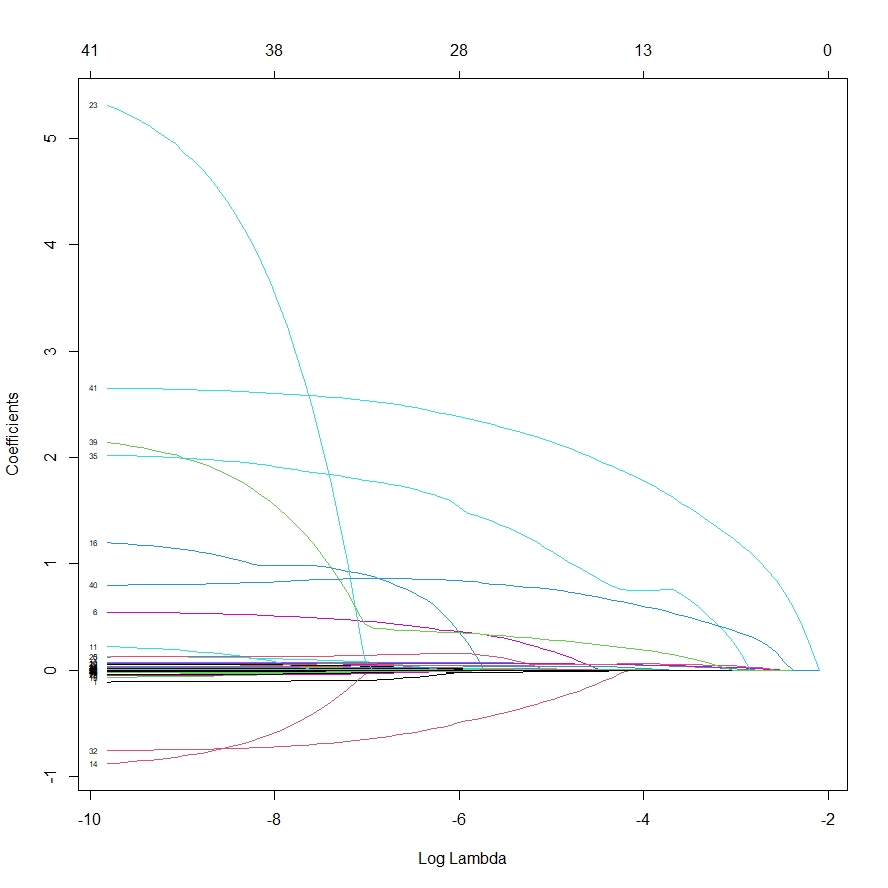

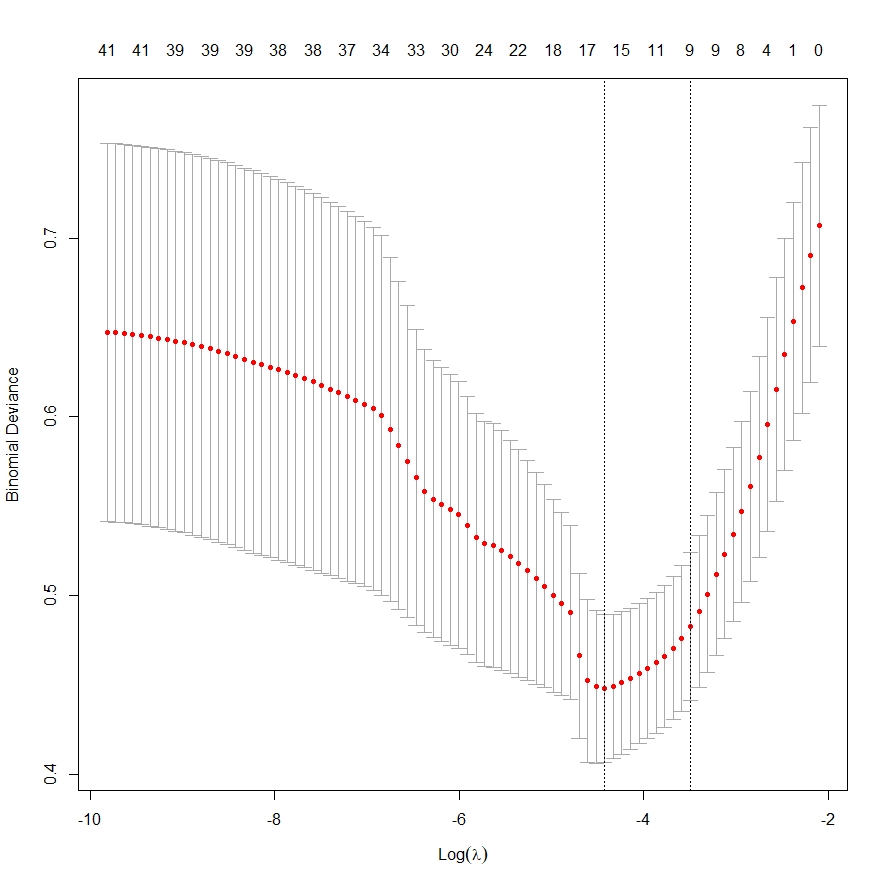


FigureS1 Penalty chart of predictive factors for severe acute pancreatitis based on LASSO regression analysis. Left: Regression coefficients. With the value of λ increasing, the absolute values of coefficients decrease. Right: Identification of the optimal λ value in the LASSO regression analysis was achieved by 5-fold cross-validation. (The left vertical line is drawn using the minimum criterion and the right vertical line is drawn using the 1_se criterion. In our study, LASSO regression model with ‘λ_1se’ criterion was used in the univariate analysis in order to solve such multiple co-linear relationships among the explanatory variables. LASSO= least absolute shrinkage and selection operator.


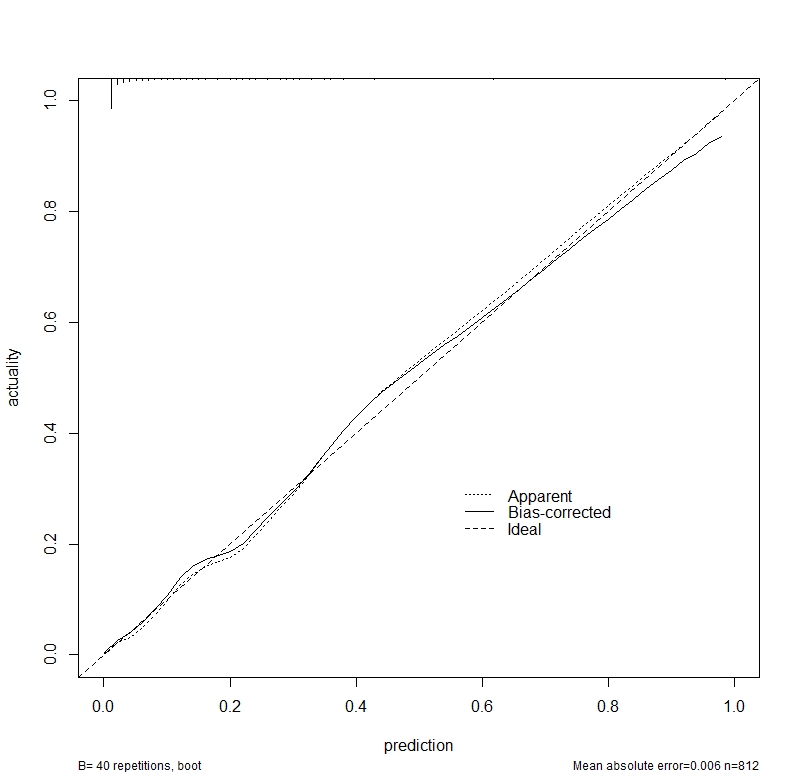

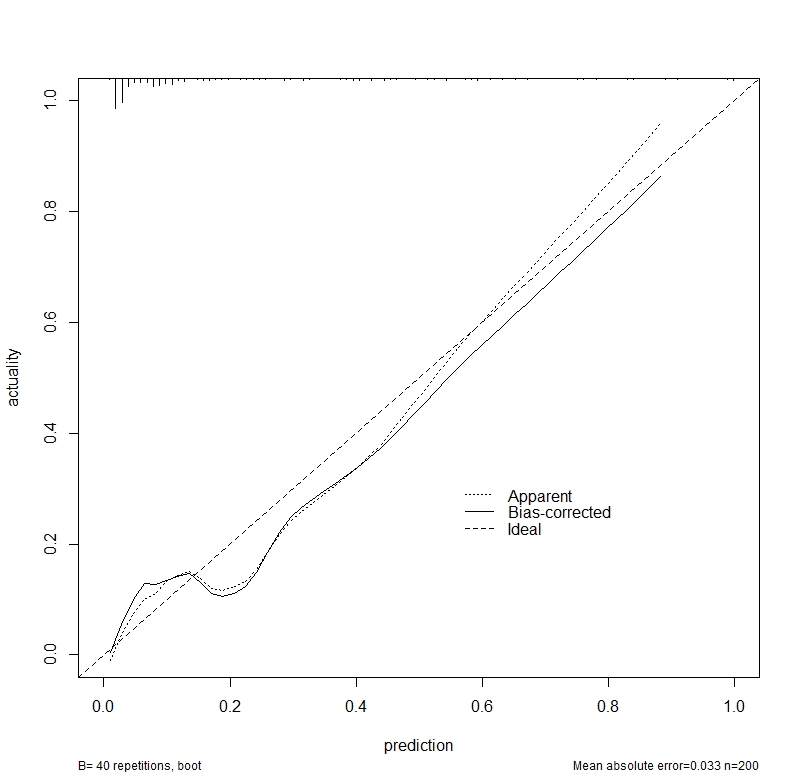

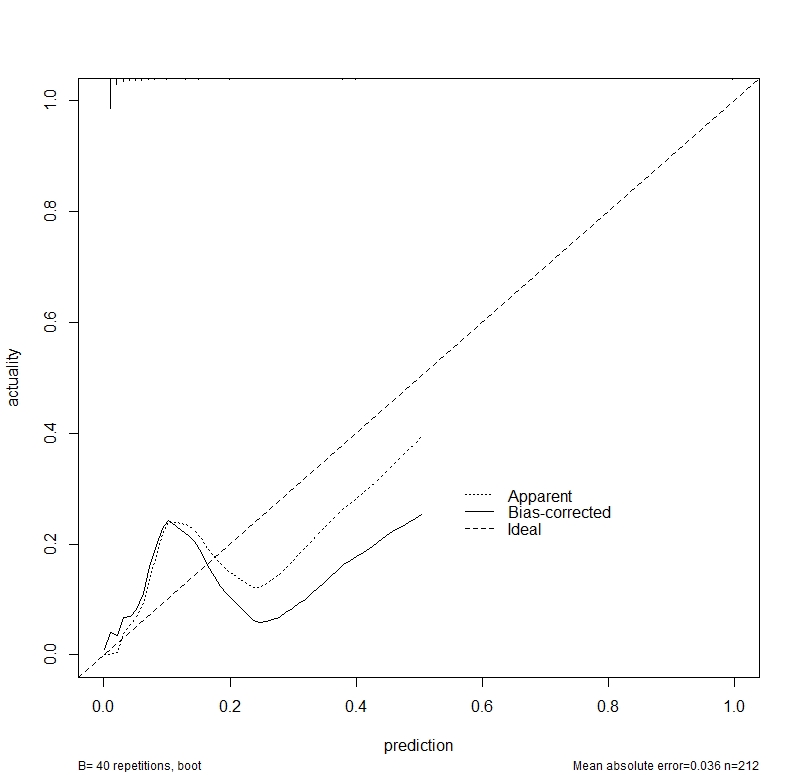


FigureS2 Calibration curve of the LASSO model in the training, validation and test set, with the mean absolute errors being 0.006, 0.033 and 0.036, respectively. The calibration curves demonstrated that the estimated risk using LASSO model was close to the observed risk, indicating a high degree of reliability.


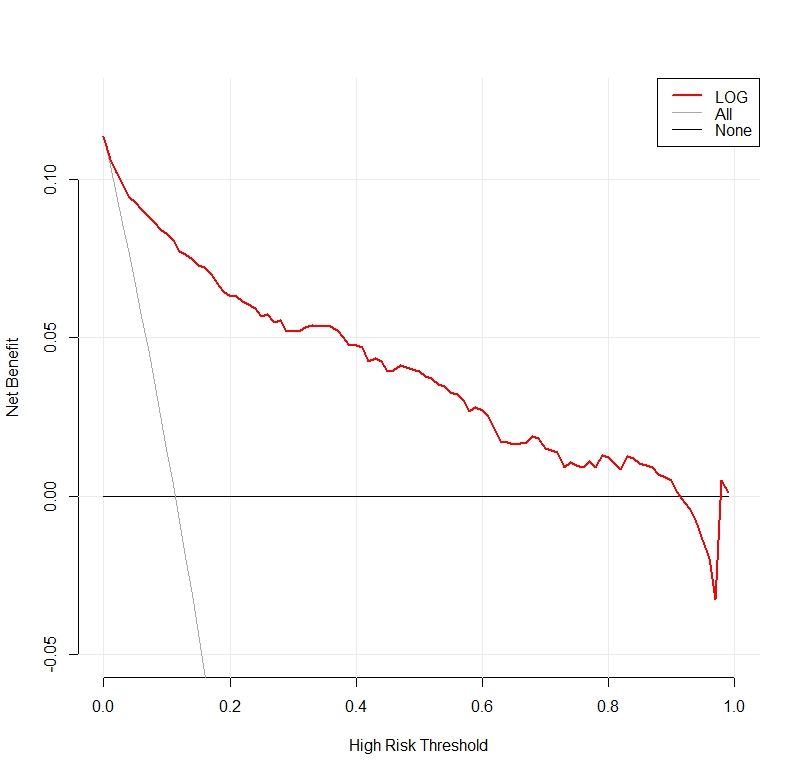

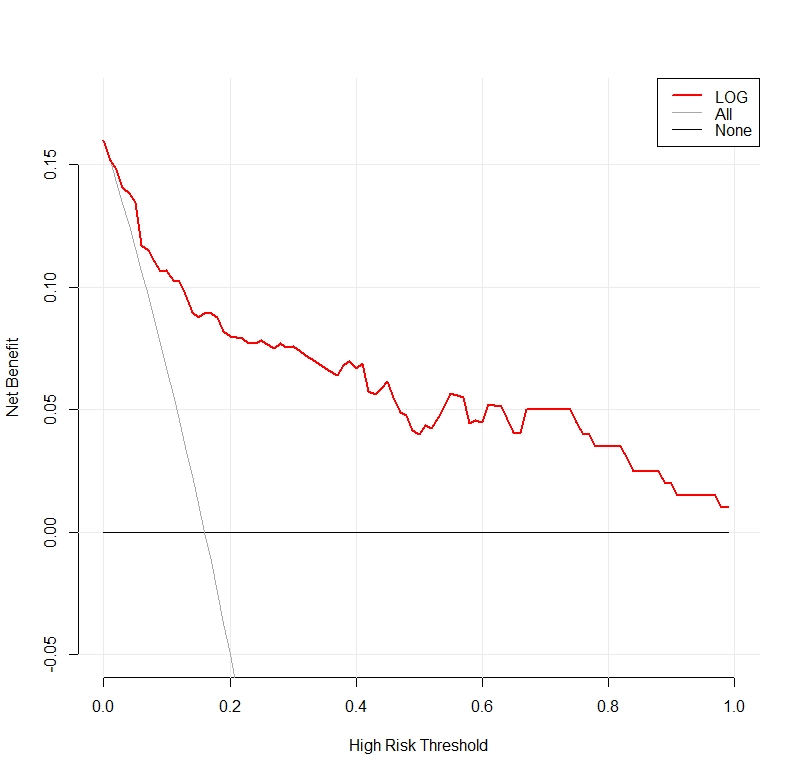

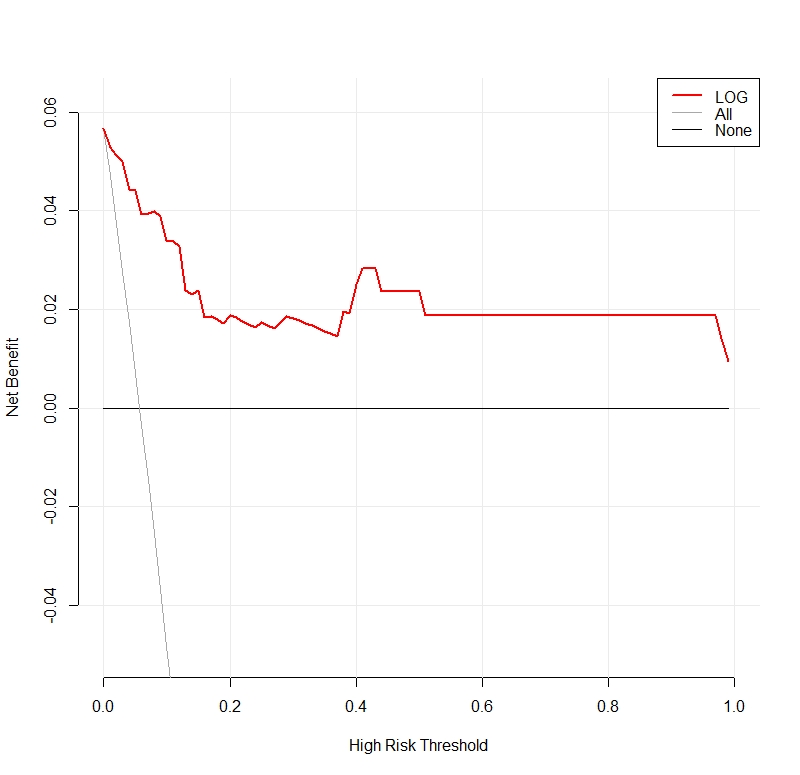


FigureS3 Decision curve analysis of the LASSO model in the training, validation and test set. The DCA plots demonstrated that when the threshold probability of SAP predicted by the LASSO model was between 10% and 100%, an intervention might add more benefit (6-10%).


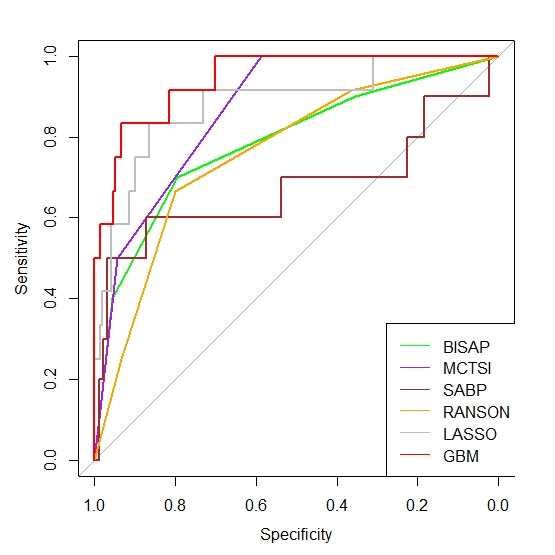


FigureS4 ROC curves of all proposed models (GBM and LASSO models) and traditional scoring systems (BISAP, MCTSI, RANSON and SABP). GBM= Gradient Boost Machine; LASSO= the Least Absolute Shrinkage and Selection Operator; BISAP= bedside index of severity in acute pancreatitis; MCTSI= modified computed tomography severity index; RANSON=RANSON score; SABP[5].


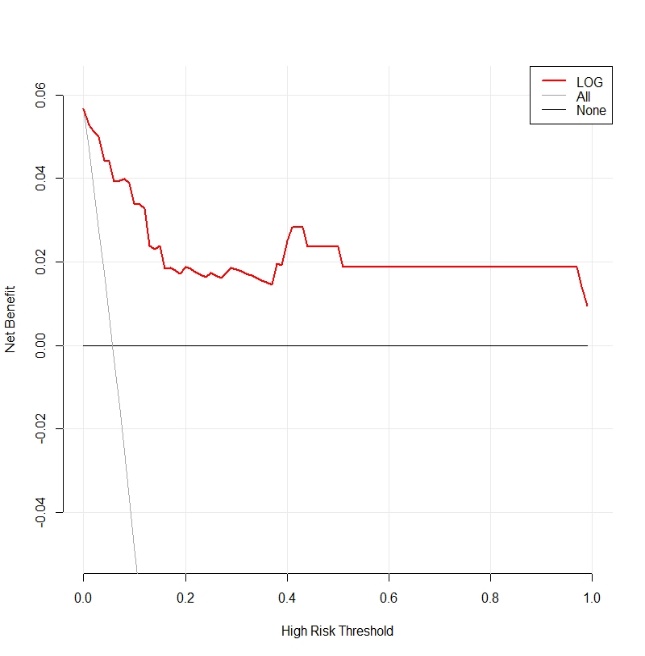

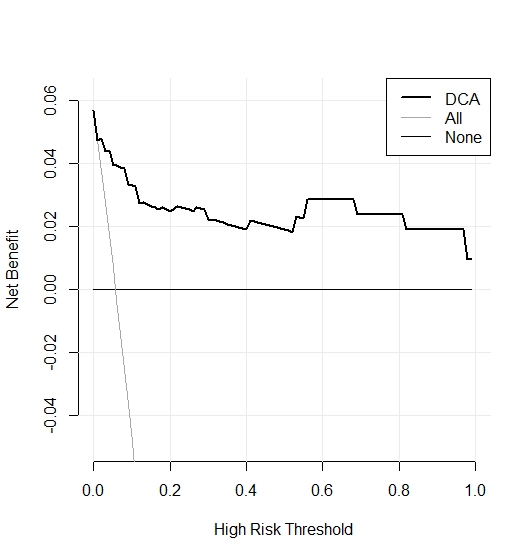

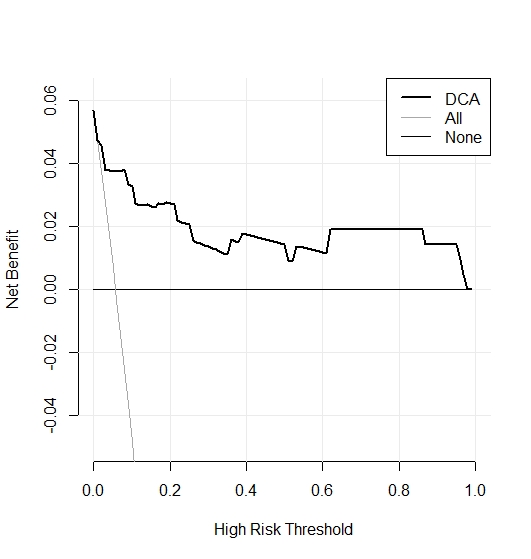

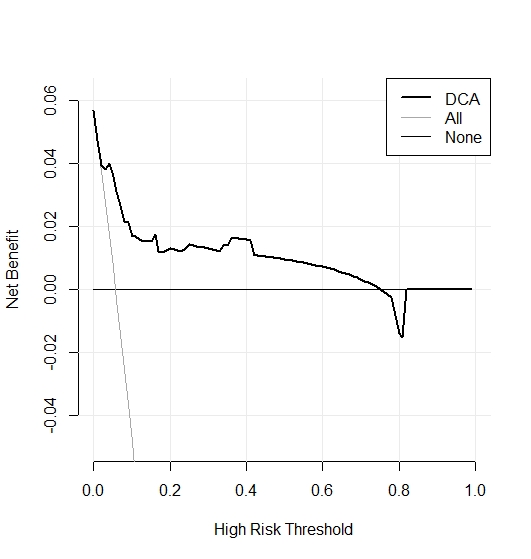

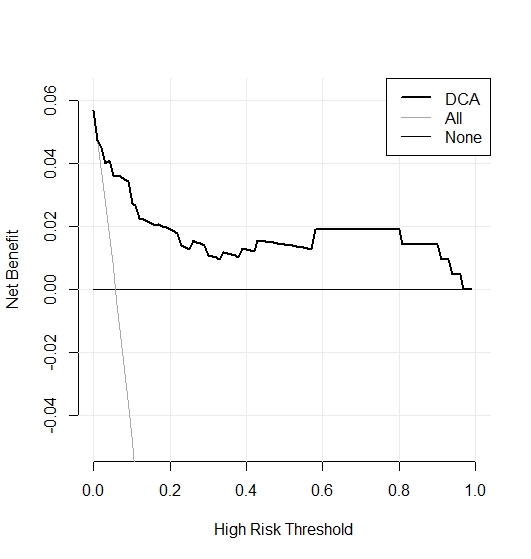

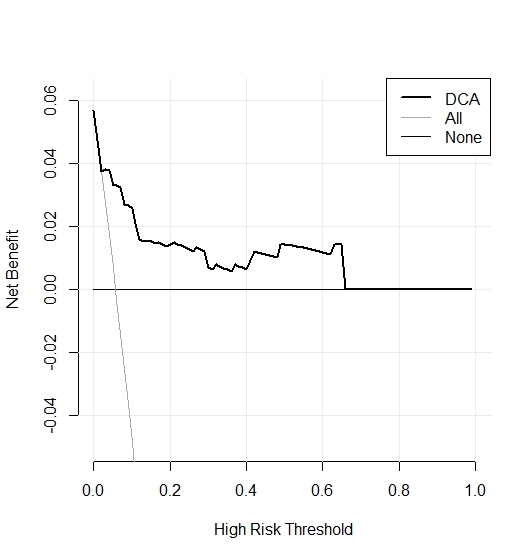


A

B

C

D

E

F

Figure S5 Decision curve analysis plots of all 6 models in the test set, indicating net benefits of around 6%. (A) LASSO model; (B) GBM model; (C) Xgboost model; (D) GLM model; (E) DRF model; (F) DL model.

Table S1. Comparison of LR and AutoML models for early predicting SAP in the validation cohort

|  |  | **AUC** | **sensitivity** | **specificity** | **accuracy** |
| --- | --- | --- | --- | --- | --- |
| **AutoML** | **GBM** | 0.942 | 0.452 | 0.995 | 0.917 |
|  | **XGBoost** | 0.933 | 0.548 | 0.984 | 0.921 |
|  | **DRF** | 0.916 | 0.581 | 0.984 | 0.926 |
|  | **GLM** | 0.912 | 0.581 | 0.957 | 0.903 |
|  | **DL** | 0.931 | 0.516 | 0.978 | 0.912 |
| **Logistic regression** | **LASSO** | 0.861 | 0.765 | 0.896 | 0.885 |
| **Existed scoring systems** | **RANSON** | 0.622 | 0.531 | 0.696 | 0.840 |
|  | **MCTSI** | 0.782 | 0.714 | 0.860 | 0.855 |
|  | **BISAP** | 0.784 | 0.625 | 0.791 | 0.765 |
|  | **SABP** | 0.784 | 0.500 | 0.745 | 0.765 |

LR=logistic regression; AutoML=automated machine learning; SAP=severe acute pancreatitis.

Table S2. Attributes and variables extracted from electronic medical records.

| Attributes | Variables | group |
| --- | --- | --- |
| Demographic information | Sex (%) | male |
|  |  | female |
|  | Age (year) (median [IQR]) | |
|  | Smoke (%) | no |
|  |  | yes |
| Etiology | Etiology (%) | biliary |
|  |  | hyperlipidemia |
|  |  | alcoholic |
|  |  | others |
| Concomitant diseases | Hypertension (%) | no |
|  |  | yes |
|  | Diabetes (%) | no |
|  |  | yes |
| Laboratory tests | MAP (mean (SD)) |  |
|  | PLT (*10^9/L) (mean (SD)) | |
|  | WBC (*10^9/L) (median [IQR]) | |
|  | N (*10^9/L) (mean (SD)) | |
|  | L (*10^9/L) (median [IQR]) | |
|  | NLR (median [IQR]) |  |
|  | HCT (L/L) (mean (SD)) | |
|  | RDW (%) (mean (SD)) |  |
|  | Lr (%) (median [IQR]) |  |
|  | PCT (%) (mean (SD)) |  |
|  | Cr (umol/L) (median [IQR]) | |
|  | TB (umol/L) (median [IQR]) | |
|  | DB (umol/L) (median [IQR]) | |
|  | DTR (median [IQR]) |  |
|  | Urea (mmol/L) (median [IQR]) | |
|  | LDH (U/L) (median [IQR]) | |
|  | Ca^2+^ (mmol/L) (mean (SD)) | |
|  | TG (mmol/L) (median [IQR]) | |
|  | GLU (mmol/L) (median [IQR]) | |
|  | TyG (median [IQR]) |  |
|  | ALT (U/L) (median [IQR]) | |
|  | AST (U/L) (median [IQR]) | |
|  | GGT (U/L) (median [IQR]) | |
|  | ALP (U/L) (median [IQR]) | |
|  | ALB (g/L) (mean (SD)) | |
|  | K^+^ (mmol/L) (mean (SD)) | |
|  | AGR (median [IQR]) |  |
|  | PT (s) (mean (SD)) |  |
|  | INR (mean (SD)) |  |
|  | APTT (s) (mean (SD)) |  |
|  | CRP (median [IQR]) |  |
|  | CAR (median [IQR]) |  |
|  | RCR (median [IQR]) |  |
| Imaging results | PE (%) | no |
|  |  | yes |
|  | SIRS (%) | no |
|  |  | yes |
| Existed scores | MCTSI (median [IQR]) | |
|  | RANSON (median [IQR]) | |
|  | BISAP (median [IQR]) | |
|  | SABP (median [IQR]) |  |
